# Strategies for managing corn crop residue in the context of greenhouse gas emissions

**DOI:** 10.1007/s11356-024-34759-9

**Published:** 2024-10-07

**Authors:** Monika Komorowska, Marcin Niemiec, Jakub Sikora, Marcin Suder, Zofia Gródek-Szostak, Atilgan Atilgan, Oleg Ovcharuk, Łukasz Lach, Rafał Kusa, Joanna Duda

**Affiliations:** 1 Department of Agricultural and Environmental Chemistry, University of Agriculture in Krakow, 31-120 Krakow, Poland; 2 Faculty of Production and Power Engineering, University of Agriculture in Krakow, 30-149 Krakow, Poland; 3 Faculty of Management, AGH University of Krakow, 30-067 Kraków, Poland; 4 Department of Economics and Enterprise Organization, Cracow University of Economics, 31-510 Krakow, Poland; 5 Department of Biosystem Engineering, Alaaddin Keykubat University, Merines Cd., Alanya, Kestel 07450 Turkey; 6 West Ukrainian National University, Kiev, 03041 Ukraine

**Keywords:** Carbon footprint, Biochar, Carbon sequestration, Slow-release fertilizers, Sustainable development, Corn production

## Abstract

Food production is one of the most important sources of greenhouse gas (GHG) emissions, both in primary production and in processing and the logistics chain. The most problematic and risky is the optimization of environmental effects in the stage of primary production. This is due to the significant influence of factors related to climate and soil that are difficult to predict. The scientific literature offers much information on the impact of crop residue management, but the context for assessing the impact of crop residue management in corn production on the carbon footprint is still unclear. The effectiveness of using organic additives like biochar, compost, corn, or straw to maintain soil productivity is well acknowledged. Information about the effects of particular crop residue management strategies on soil carbon sequestration, soil quality, and crop yield in corn cultivation is currently scarce. The research aimed to assess the potential for optimizing corn production through modifications in crop residue management, with a focus on the efficiency indicator being the level of greenhouse gas emissions per functional unit of the product. A 3-year growing experiment was conducted to investigate the impact of different corn crop residue management strategies. The modifications of the corn cultivation technology in terms of the crop residue management strategy had a significant impact on the yield of plants and the amount of GHG emissions. The conversion of corn straw to biochar and its introduction into the soil reduced the GHG emissions from corn cultivation per functional unit, despite the energy expenditure related to straw transport and biochar production. From a 3-year time perspective, a beneficial effect of biochar addition on the size of the commercial yield of plants was observed. In variants with biochar and a reduced level of nitrogen fertilization, no reduction in yields was observed. This confirmed the hypothesis that biochar could be a useful material for the production of slow-acting fertilizers.

## Introduction

The objectives of sustainable energy development entail environmentally friendly management of production processes. Plant production stands out as one of the key sources of greenhouse gas (GHG) emissions (Wang et al. 2022). GHG emissions in primary production are contingent upon the agronomic efficiency of the means of production employed and the plant yield. During crisis conditions that hinder the growth and development of plants, such as droughts, the level of GHG emissions per functional unit of the product notably rises (Chowaniak et al. 2020; Sikora et al. 2020a). Johnson et al. (2013) indicate that the most important factors related to the environmental efficiency of biomass use include the type of raw material, geographic location, land use change, and the time perspective and the related change in the soil carbon dioxide binding potential. In the case of using waste biomass from various industries, the source of GHG emissions could be its processing and refining (Niemiec et al. 2017; Akbari et al. 2021). Activities related to the reduction of GHGs from primary production are of strategic importance for the development of energy biomass production. Greenhouse gas emissions from agricultural production are associated with the production and use of mineral fertilizers and pesticides, fuel combustion, and the manufacturing of agricultural machinery. In conditions of water scarcity, irrigation represents a significant source of greenhouse gas emissions. This source could even constitute up to 50% of the total GHG emissions in dry areas due to inefficient irrigation technology (Ishimoto et al. 2018; Rashidov et al. 2021).

An important source of GHGs is the emission of nitrogen oxides from nitrogen used for fertilization. Unused nitrogen from mineral and organic fertilizers is an important source of GHG from agricultural production. Nitric oxide (N_2_O) has a potential approximately 300 times greater to generate the greenhouse effect. The mineralization of organic matter is a very important aspect related to the production of plants. The loss of organic matter in the soil is the result of intensive cultivation and aeration of the soil, as well as intensive fertilization.

In agronomy, the most efficient approach to reduce the carbon footprint of plant production is to enhance the nitrogen use efficiency from mineral fertilization (Niemiec et al. 2015; Chowaniak et al. 2020; Sikora et al. 2020a; Shin et al. 2021).

An additional approach to enhance the environmental sustainability of agricultural crops involves reducing GHG emissions from soil resources. Organic matter depletion stands out as one of the primary causes and outcomes of soil degradation. This occurs due to intensive soil cultivation, fertilization, and alterations in soil pH. Soil serves as a vital carbon reservoir and plays a crucial role in the biogeochemical cycle of this element (Agegnehu et al. 2017). The cultivation of plants in areas with a high carbon sequestration potential, such as permanent meadows, peat bogs, forests, and natural areas with grassy vegetation, is particularly harmful in terms of the impact of agriculture on the greenhouse effect. In the long term, the production of energy crops in such areas is usually unjustified from the point of view of climate change (Rahman et al. 2021).

An important issue from the point of view of GHG emissions is crop management of plants such as rape, corn, and wheat that produce a large amount of residue with significant amounts of organic carbon and a high energy potential. From the point of view of energy production, it is rational to use straw, for example as a combustion fuel. However, proper crop residue management is of strategic importance when it comes to assessing GHG emissions from agricultural production and maintaining soil fertility (Cui et al. 2021). Depending on the method of introducing straw into the soil ecosystem, different production results are obtained, which are related to the rate and direction of its decomposition. Islam et al. (2022) showed that with high nitrogen fertilization and deep cultivation, leaving wheat and corn straw in the field can have positive production effects. Improper crop residue management reduced the yield of subsequent crops (Islam et al. 2022). The processes of straw mineralization in the soil lead to the emission of a significant amount of carbon dioxide and use up nitrogen available in the soil, which could reduce the amount available to plants (Jiang et al. 2022). From the point of view of carbon sequestration and modeling soil properties, this could be ineffective. The contemporary approach to the management of soil resources focuses on reducing the emission of carbon compounds from the soil, e.g., through measures supporting the permanent sequestration of this element in the soil.

Organic materials introduced into the soil or left as crop residue undergo various transformations, the most important of which are mineralization and humification. The intensity and direction of these changes depend on many factors, both natural and environmental, for example, soil reaction, climatic conditions, cultivation intensity, the level of nitrogen fertilization, or the use of irrigation (Sousa Lima et al. 2018). In terms of the transformation of carbon compounds, the ratio of carbon to nitrogen in the soil is very important. These elements are responsible for microbiological transformations of organic matter since as a result of mineralization, nutrients are released, which increases the yield.

Organic matter profoundly impacts soil fertility, environmental functional parameters, mechanical and thermal properties, buffering capacity, sorption complex capacity, and air–water relations. Increasing organic matter content is crucial for optimizing soil resource usage. Efficient management of soil organic carbon is indispensable within all quality management systems, including the GLOBAL GAP standard, SAI Platform, and various national or private systems. Additionally, the proliferation of specific microorganism species can bolster humification processes, potentially leading to the permanent sequestration of organic carbon (Finn et al. 2017; Hou et al. 2021).

The conversion of biomass provides opportunities to enhance carbon sequestration upon its incorporation into the soil. The strategic goal of converting carbon into biochar is to mitigate agriculture’s impact on intensifying the greenhouse effect. The scientific literature broadly discusses the issue of using biochar as an additive to the soil, but most authors ignore issues related to the collection of biomass, its transport to the treatment site, and energy consumption and greenhouse gas emissions during the torrefaction process (Gross et al. 2022; Agegnehu et al. 2017).

Corn is a plant with a high yield potential; therefore, it is a globally important fodder and consumption crop. The plant makes good use of water and solar radiation, but production of its crop is accompanied by a significant consumption of production resources. This negatively affects the level of impact of this plant’s production on the greenhouse effect (Wang 2021). Optimizing the production of this crop to reduce GHG emissions should be a priority in agricultural sciences (Jia et al. 2020; Costantini and Bacenetti 2021).

The aim of the investigation was to evaluate the possibilities of optimizing corn production by modifying crop residue management. The adopted research hypothesis is that the torrefaction of the crop residue of grain corn and the production of fertilizers based on biochar and a nitrogen valorizing additive will:

- increase the yield of at a constant level of fertilization
- reduce GHG emissions from cultivation per functional unit of the product

## Material and methods

### The design and location of the experiment

To reach the adopted goal, a 3-year growing experiment was carried out, in which the experimental factor was the strategy of corn crop residue management. The experiment was located on a commodity farm in Warminsko-Mazurskie Province, in the north-eastern part of Poland (20.482770 N, 53.871265 E). The test plant was corn, *Zea mays* L., cultivar Dekalb 3609. The growing experiment was conducted in the years 2019–2021, and the plants were grown in monoculture. The experiment was set up on November 7, 2018 when organic materials were introduced to the soil before sowing. The forecrop was corn, and the experiment began with the management of forecrop residues. The forecrop yield was 9.96 t/ha, and the amount of forecrop residues was 13.74 t/ha. The seeds were sown on the soil with the grain composition of heavy clay (Table 1).

**Table 1:** The properties of the soil used for the experiments

pH in H_2_O	pH in KCl	N total	C org	N min	P	K	Mg	Ca
		(g/kg)		(mg/kg)				
6.78	5.22	0.179	1.72	48.76	164.7	288.2	115.8	641.3

In the conducted experiment, the research factor was the direction of crop residue management. The experiment design is given in Table 2.

**Table 2:** The experiment design

Object designation	The method of crop residue management	S	N	P_2_O_5_	K_2_O
A	Crop residue removal	40	250	97	240
B	Leaving raw crop residue in the field	40	250	97	240
C	Biochar torrefaction and introduction into the soil	40	250	97	240
D	Biochar torrefaction and introduction into the soil	40	200	97	240
E	Introduction of biochar-based fertilizer	40	250	97	240
F	Introduction of biochar-based fertilizer	40	200	97	240

The field experiment included an object in which the crop residue was removed (A) and an object in which the crop residue was left (B). In the third and fourth objects (C and D), the biochar applied was produced from the secondary crop with variable nitrogen fertilization. In the next object, a mineral-organic fertilizer based on biochar and urea was used (E and F) (Table 2). In the object where biochar was introduced, it was applied before sowing and then mixed with the soil. Biochar was applied in the form of granules, and fertilization was carried out according to the fertilization needs of plants, calculated based on nutritional requirements, soil properties, climatic conditions in the research area, and historical data on the yield of the test plants. Seventy thousand plants were sown per hectare. For objects where biochar was used, nitrogen fertilization was reduced to assess the ability of biochar to reduce nutrients. The field experiment was conducted in four replications using the randomized block method. Prior to commencing the experiment, the habitat’s production potential was estimated at 11.5 Mg of dry seeds/ha. Soil samples were collected for basic agrochemical analyses. Soil pH was determined using the potentiometric method, while mineral nitrogen was assessed using the distillation method following extraction with a 1 Mol/dm^3^ solution of potassium sulfate. Total nitrogen and organic carbon were analyzed using the Vario Max Cube by Elementar. Assimilable phosphorus and nitrogen were determined using the Egner-Riehm method and calcium and magnesium using the Schaschsabela method. The concentration of elements in the solutions was determined using inductively coupled plasma optical emission spectrometry (ICP-OES) with the Optima 7600 DV device by Perkin Elmer. The soil properties are detailed in Table 1. Fertilization technology was designed based on the soil analysis results. Nitrogen was applied in two doses, with 70% applied before sowing and the remaining 30% applied as top dressing during the developmental phase, at five to seven leaves. Urea was used for pre-sowing, while potassium nitrate was used as top dressing. A single herbicide, Cornmax 340SE (Bromoxynil 90 g/l terbutylazine 250 g/l), was applied at a rate of 1 l/ha during cultivation. The results of meteorological observations are presented in Table 3.

**Table 3:** Average monthly temperatures (°C) and rainfall sum (mm) in individual months during the study period

Year	I	II	III	IV	V	VI	VII	VIII	IX	X	XI	XII
2019	0.2	3.8	7.2	11.2	12.4	22.7	20.3	21.2	15.2	11.5	7.6	4
2020	2.8	2.5	5.8	10.6	12.4	18.4	19.3	21.1	15.9	11.4	6.3	2.8
2021	0.1	0.1	4.5	6.8	12.7	20.5	21.1	18.1	15.8	10.5	5.6	1.2
Rainfall												
2019	42.9	26.4	28.5	38.1	60.2	27.2	49.3	44.2	58.4	30.2	33.2	13.9
2020	7.6	60.9	12.7	12.4	84.4	210.9	42	77.2	93.1	91.4	19.5	14.4
2021	38.4	18.5	18.5	42.7	62.5	45.2	90.4	89.2	26.2	9.4	27.6	32.5

### Properties of biochar used in the experiment

Corn crop residues were transformed into biochar through a 20-min pyrolysis process at 450 °C. The torrefaction process parameters and the biochar properties are detailed in Table 4.

**Table 4:** Key parameters of the torrefaction process and the biochar employed in the experiment

Following parameters	Unit	Value
Temperature of the torrefaction process	°C	450
Duration of the torrefaction process	Minute	20
Efficiency of the torrefaction process	% by weight of the raw material	41.1
CO_2_ emissions related to the production of biochar	kg CO_2_ eq Mg^−1^ of biocha	170
Organic carbon content	%	82.79
Stable carbon content in biochar	% of total carbon	77.42

The efficiency of biochar production in the applied process parameters ranged from 39 to 43% of the mass of crop residues used in the study. Biochar parameters are as follows: specific surface area 84.1 (m^2^G^−1^), pH KCl 9.1, bulk density 0.15 g/cm^3^ ash content 33.4%, and organic carbon content 68.4%. In terms of porosity, the parameters are as follows: average pore radius (0.24 μm), total pore area (18.14 m^2^/ g), total porosity (75.92%). Biochar properties were assessed using scanning electron microscopy (SEM) via the Zeiss Ultra Plus apparatus, manufactured in Germany, at 5 kV. Sample preparation for SEM involved fine grinding.

### Properties of the biochar- and urea-based fertilizer

As part of the optimization of the use of biochar as an additive to the soil, a formula for a fertilizer containing biochar and urea was developed. The fertilizer was made of biochar from harvested corn straw. The share of urea in the fertilizer was 21.7%. The nitrogen content in the fertilizer was 10%. The electricity consumption for the production of 1 t of granulated fertilizer based on urea and biochar required 15.3 kWh of electricity.

### Environmental impact assessment methodology

The level of environmental impact of corn production in various production variants was determined using ISO 14040: “Environmental Management—Life Cycle Assessment—Principles and framework” and ISO 14044: “Environmental Management—Life Cycle Assessment—Requirements,” as well as the recommendations in the document (ILCD 2010). The analysis did not encompass the production of seeds, their transportation, or agricultural tools. The functional unit was set at 1 ton of commercial product, and the analysis of a single system spanned 1 year. Greenhouse gas (GHG) emissions were estimated based on carbon dioxide (CO_2_) equivalent. Data for GHG emissions were sourced from six experimental objects with differing crop residue management strategies. The emission level of nitrogen resulting from the production of ammonium nitrate was set at 7.99 kg CO_2_/kg N. The greenhouse gas emissions resulting from the production of potassium chloride and triple superphosphate were 0.56 and 0.36 kg CO_2_/kg nutrient, respectively (Kool et al. 2012). Ammonia emissions to the atmosphere were calculated following guidelines by Nemecek and Schnetzer (2012). The emissions associated with the decomposition of crop residues were determined based on the quantity of crop residue in corn cultivation. Experimental results estimated that the ratio of marketable dry crop residue to harvest residue in corn cultivation ranged from 0.72 to 0.83%. The carbon fraction in the dry matter of the harvest residue ranged from 44.16 to 49.38%, and the nitrogen content in the harvest residues ranged from 0.74 to 0.86% in dry matter. Harvest residue decomposition was estimated at 25% based on an incubation experiment (unpublished data). Input data for GHG emissions from individual production areas are detailed in Table 5.

**Table 5:** Input data sources for calculating GHG emissions

No	Emission source	Data source
1	Amount of emissions from crop residue	IPCC (2006)
2	N-N_2_O emission related to the transformation of nitrogen compounds in the soil	Novoa and Tejeda (2006), IPCC (2006)
3	CO_2_ emissions from the production of mineral fertilizers	Kool et al. 2012
4	CO_2_ emissions from diesel combustion	EPA (2016)
5	GHG emissions related to the production of pesticides	Audsley et al. (2009)
6	Energy consumption for drying corn	Torrez, Irigoyen, and Giner (2016)
7	Emissions related to the production of electricity	Wang et al. (2021)
8	N–N_2_O emission from mineral fertilization	FAO (2017)
9	Fuel consumption for agrotechnical treatments	Wójcicki (2015)

Greenhouse gas emissions as carbon dioxide and nitrogen oxides from crop residues in the soil were calculated using input data contained in the IPCC (2006). According to Novoa and Tejeda (2006), 1.25% of the nitrogen contained in the post-harvest biomass is emitted as N_2_O. N_2_O has a 292 times greater potential for generating the greenhouse effect than carbon dioxide (Forster et al., 2007). The mineralization index of soil organic matter was set at 1.6%. Additionally, the emission values of nitric oxide resulting from nitrogen transformations in the soil were taken into account following the IPCC methodology (2006). In the life cycle assessment (LCA) of corn, greenhouse gas emissions associated with the use of diesel for soil cultivation and crop harvesting were considered. Detailed fuel consumption data for individual agricultural operations can be found in Table 6.

**Table 6:** Greenhouse gas emissions and the associated energy consumption in agrotechnical treatments

Description	Unit	Diesel consumption	Energy consumption	CO_2_ eq emission
Unit		dm^3^	Mj	kg
Tillage	dm^3^ of diesel/ha	21	850.5	81.5
Mineral fertilization, 2 treatments	dm^3^ of diesel/ha	4	162.0	15.5
Cultivation with an aggregate	dm^3^ of diesel/ha	25.1	1016.6	97.4
Corn sowing	dm^3^ of diesel/ha	22.1	895.1	85.7
Crushing crop residue	dm^3^ of diesel/ha	11	445.5	42.7
Mechanical harvesting	dm^3^ of diesel/ha	22	891.0	85.3
Transport of grain to the farm	dm^3^ of diesel/1 t km^−1^	0.51	20.7	2.0
Drying corn	dm^3^ of diesel/Mg of grain/°C	7.4	299.7	6.8413
Transport of biochar from the processing site	dm^3^ of diesel/t/30 km	2.1	85.1	8.1
Transport of straw to the processing site	dm^3^ of diesel/t/30 km	2.2	89.1	8.5
Application of plant protection products	dm^3^ of diesel/ha	2.5	101.3	9.7
Mechanical harvesting of straw	dm^3^ of diesel/ha	22.1	895.05	85.73276
Biochar application	dm^3^ of diesel/ha	11	445.5	42.67241
Manufacture of biochar-based fertilizers	kwh/t of fertilizer	15.3	55,08	14.14

Fuel consumption for each treatment was calculated based on data from Wójcicki (2015). The amount of carbon dioxide associated with diesel combustion was set at 3.864 kg CO_2_/dm^3^ (EPA 2016). The emission of nitrogen oxides resulting from diesel engine operation was excluded from the system boundary as recommended by the EPA (2016). The CO_2_ emission resulting from the production of 1 kg of active substance in pesticides was set at 25.5 kg CO_2_ (Audsley et al. 2009) (Table 4). Energy consumption for drying corn grain was based on Torrez Irigoyen and Giner (2016). Corn seeds, harvested with moisture levels ranging from 19.82 to 24.46%, were dried to 14% moisture content using an SPG batch dryer powered by fuel oil. The average fuel oil consumption for drying was 67 MJ/t/°C. Biochar production process energy was sourced from electricity, with associated CO_2_ emissions of 170 kg CO_2_ eq/t of biochar. In European conditions, the production of 1 kWh of electrical energy generates emissions of 0.9245 kg CO_2_ (Wang et al. 2021). According to FAO (2017), approximately 1% of nitrogen introduced into the soil from mineral fertilizers is emitted directly as nitrogen oxides, while an additional 0.27% is released into the atmosphere as indirect emissions. Furthermore, about 0.75% of nitrogen unused by plants undergoes emission into the atmosphere due to chemical and biological transformations (FAO 2017). The life cycle assessment for the production of biochar from crop residues was calculated as 1 Mg of biochar. The cultivation site was approximately 30 km from the torrefaction site, as per the adopted research objective. The structure of the experiment was developed based on the established research objective, taking into account the risk of factors that could disrupt the accuracy of inferences and the risk of analytical errors (Montgomery 1991). The risk analysis was conducted in accordance with the guidelines of ISO 31000:2018.

**Fig. 1:**
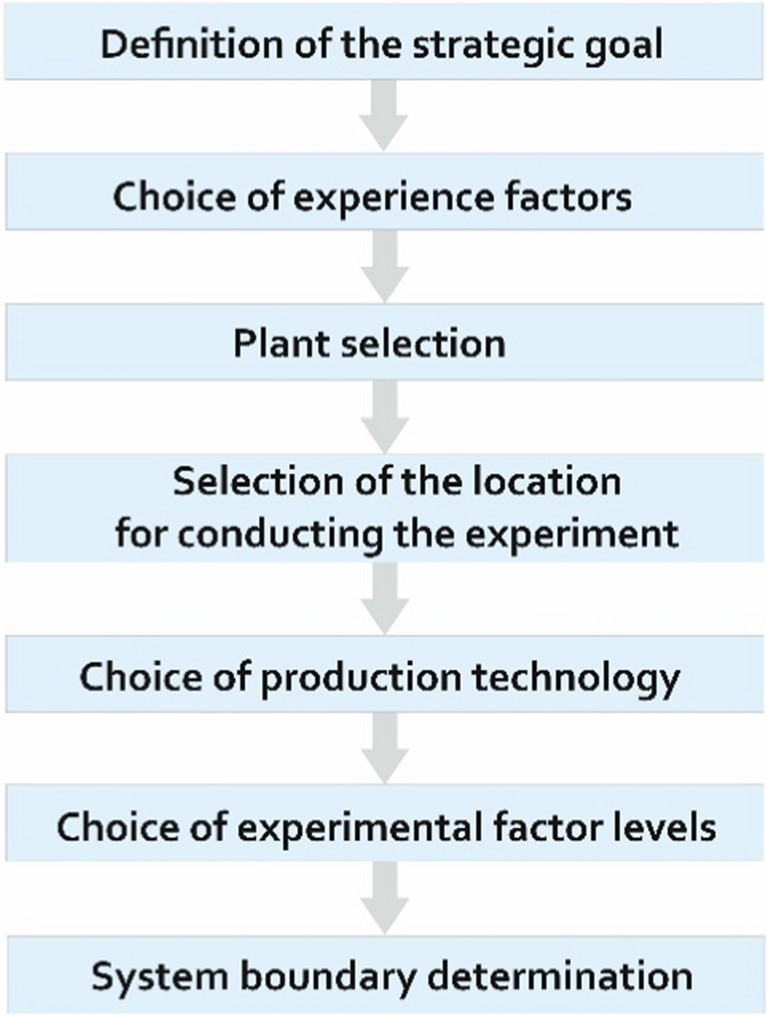
Experimental scheme

The experiment was carried out according to the following Fig. 1.

**Fig. 2:**
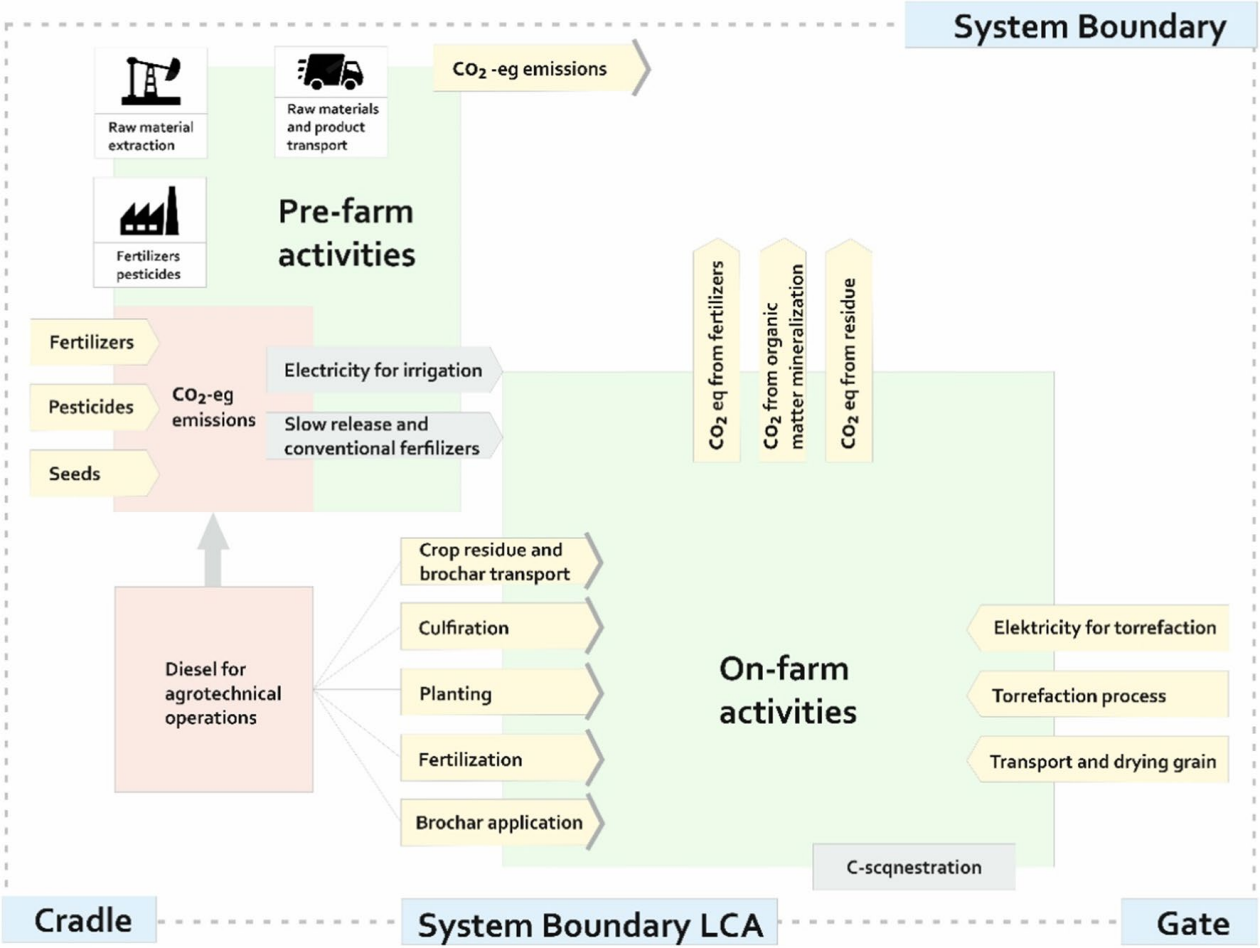
Boundary system

The system boundaries (Fig. 2) included:

1. Production of fertilizers used to grow plants
2. Energy consumption for field work on the farm
3. Soil emissions (direct and indirect) from fertilizer use
4. Emissions from the management of crop residues and emissions from soil organic matter mineralization
5. Emissions related to the production of biochar both in terms of energy consumption and GHG emissions during the process
6. Emissions related to the production of seed material and plant protection products
7. The emission of methane from the cultivated area was excluded from the boundary of the system, because it is of marginal importance. Under the adopted experimental conditions, it can be expected that it will take on negative values (Pareja-Sánchez et al. 2019)

### Statistical methods

Both basic and advanced statistical methods were used in the statistical analysis of the data described above. To verify the differentiation of the studied distributions, a standard test of independence was used, based on the chi-square statistics (Zeliaś, 2000). To verify the significance of the variation in average CO_2_ eq emissions for individual variants, taking into account the time factor, repeated measures ANOVA was used, which is applicable in medicine and in agricultural sciences (Stanisz 2007). In all statistical tests, the standard significance level was adopted, i.e., 5%. The analysis was performed with the use of Statistica 13 software.

## Results and discussion

The results of the experiment show that the method of organic matter management and the use of slow-release fertilizers impacted plant yields. In the 3-year period, the yield of corn ranged from 6.15 to 11.86 t/ha. Significant differences in yielding were found in individual years. On average, the lowest yield was obtained in 2019 due to insufficient rainfall during the growing season (Table 7).

**Table 7:** GHG emissions in individual objects, per experiment years, in CO_2_ eq /t grain

	Total emission	Fertilizers and seeds and plant protection product	Emission from the mineralization of crop residues	Production and use of biochar	Mineralization of soil organic matter	Diesel combustion
2019						
A	1364.51	1044.19	103.27	0.00	60.90	156.14
B	1417.00	834.95	403.63	0.00	49.02	129.41
C	852.64	542.35	98.54	61.50	32.55	117.71
D	895.38	551.36	104.06	64.94	41.01	134.01
E	896.07	561.59	107.21	70.01	33.67	123.60
F	739.53	411.69	108.79	70.19	31.15	117.71
2020						
A	811.43	562.47	103.27	0.00	33.71	111.98
B	1053.00	515.53	403.63	0.00	30.98	102.86
C	777.45	474.40	98.54	61.50	28.71	114.29
D	658.13	350.52	104.06	64.94	26.97	111.64
E	695.72	389.13	107.21	69.11	23.93	106.34
F	630.19	318.47	108.79	69.70	24.64	108.59
2021						
A	884.31	625.95	103.27	0.00	37.29	117.8
B	1114.36	569.38	403.63	0.00	34.02	107.3
C	830.13	520.38	98.54	61.50	31.31	118.4
D	708.75	393.22	104.06	64.94	29.95	116.6
E	788.99	469.47	107.21	69.53	28.46	114.3
F	709.73	384.94	108.79	70.04	29.28	116.7

The average yield for all objects was 7.48 t/ha. The highest yields were obtained in 2021, at 10.23 t/ha. In the first year of research, the highest crop of plants was obtained in objects where biochar and fertilizer based on urea and biochar (variants C, D, E, F) were used. One of the most important positive aspects of using biochar as a soil additive is improving the efficiency of water use in light of the current water shortage in Poland. The year 2019 was characterized by a serious water deficit; therefore, the introduced biochar could increase the efficiency of water use, as confirmed by the research of Seyedsadr et al. (2022). Even in the case of surface introduction of biochar, a positive effect on the availability of water and nutrients for plants was observed (Gao, De Luc, 2021; Huang et al., 2021a, b). The results presented by these authors indicate the possibility of effective use of biochar on permanent lands, which broadens the prospects for the use of biochar in agriculture in the context of increasing soil water sorption and improving its fertility. In turn, Liang et al. (2021) found no unequivocal influence of biochar on soil fertility. These authors proved the positive effect of leaving straw in the soil on plant yields and soil properties. However, they did not find any difference between the variants where straw, straw compost, and straw biochar were used. In the presented research, the highest average yield over 3 years was obtained in the objects where fertilizer based on urea and urea was applied. There was no statistical difference between objects E and F, despite the reduction of nitrogen dose by 20% in object F. The obtained results prove the ability of biochar to reduce nitrogen losses and increase the efficiency of nitrogen fertilization. Similar results were obtained by other authors such as Krause et al. (2018), Jin et al. (2019), and Hu et al (2021).

The use of biochar on agricultural land improves soil fertility, which consequently increases the use of nutrients, water, and microelements contained in the soil. The reduction of GHG emissions from the use of biochar is related to the sorption of carbon dioxide in soil air and the reduced emissions of nitrogen oxides, which are considered the most important agricultural GHGs (Krause et al. 2018). Gross et al. (2022) found that the addition of biochar to the soil increased its bioaccumulation in the soil. These authors unequivocally state that the use of biochar limits GHG emission from agricultural production. In their research, they proved that biochar increases the level of carbon accumulation in soil. Two years after the use of 7 Mg C/ha of biochar, carbon sequestration from the atmosphere increased to approximately 2.5 Mg/ha. In addition, these authors found approximately 21% lower GHG emissions compared to the object fertilized with an equivalent amount of manure. These studies did not take into account the biochar production system boundary and the transport of biomass and biochar from their production sites to agricultural production sites. Anand et al. (2022) calculated that the introduction of 1 Mg of biochar to the soil reduces the loss of fertilizing elements by 4 kg. The reduction of the losses of fertilizing elements results from the high sorption capacity of biochar. Awad et al. (2018) observed that incorporating the mineral fraction into biochar, using components such as oyster shells or polymer compounds, notably enhanced its capacity to mitigate greenhouse gas emissions from agricultural ecosystems. Xu et al. (2016) highlighted that biochar’s CO_2_ sorption capacity can reach 34 mg/g of biochar, dependent on metallic element content. Shin et al. (2021) and Oliveira et al. (2017) independently found that biochar addition significantly increased carbon sequestration and intensified humification processes, resulting in reduced carbon dioxide emissions and enhanced soil properties. Biochar’s impact on soil microorganism communities and chemisorption of carbon dioxide were also noted to contribute to decreased emissions (Zhang et al. 2016; Yang et al. 2021). Managing soil to curtail carbon emissions and promoting long-term carbon accumulation are crucial in natural resource management. Biochar is recognized as a sustainable soil carbon source and an effective means of carbon sequestration (Jiang et al. 2022). Furthermore, Xiong et al. (2021) demonstrated that torrefaction of straw can enhance soil organic matter management, albeit with the potential for increased organic matter outflow and surface runoff, necessitating thorough soil mixing upon biochar introduction.

**Fig. 3:**
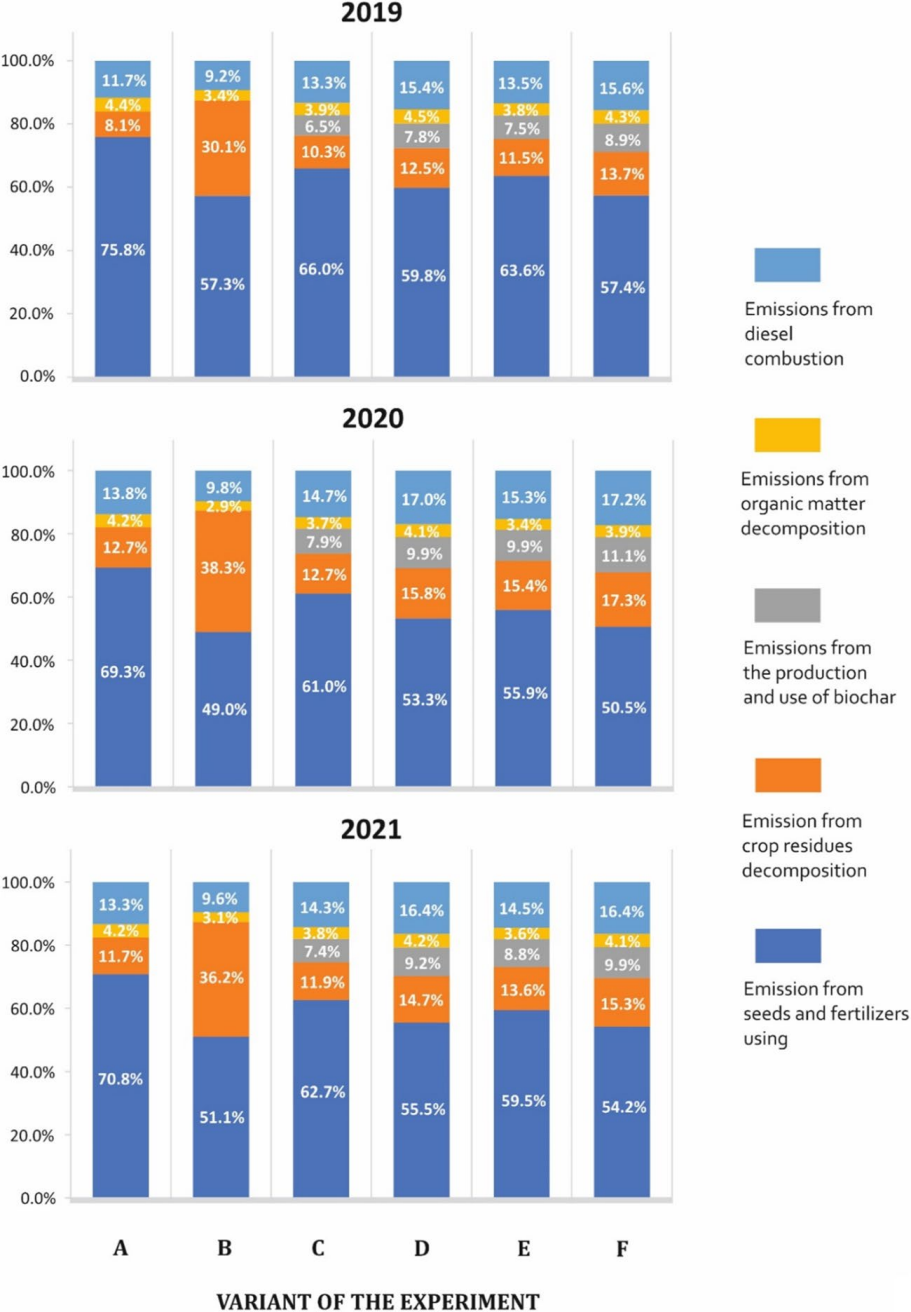
Distribution of CO_2_ emissions depending on the experiment variant. Average emission value analysis depending on the method and time (year)

The emission of CO_2_ equivalent ranged from 630.2 to 1339.8 kg of CO_2_ per ton of marketable corn yield within the adopted system boundary. This parameter’s value was mainly dependent on the yield size, which was primarily influenced by the research year. On average, over subsequent years of research, the values were 965.9, 771.0, and 839.4 CO_2_ eq/t of marketable corn yield. Throughout all the years of research, the highest level of greenhouse gas emissions was observed in the scenario where the straw remained in the field. Corn grain production in this scenario was linked to GHG emissions ranging from 1052 to 1339 kg of CO_2_ eq/t of corn grain. Removing straw from the field reduced GHG emissions by up to 25%. To conduct a more comprehensive analysis of the influence of implemented technological adjustments on the evolution of greenhouse gas emissions from corn production, the sources were categorized into five groups: emissions associated with diesel combustion, emissions linked to soil organic matter mineralization, emissions tied to biochar production and usage, emissions associated with crop residue mineralization, and emissions related to the usage of fertilizers, seeds, and plant protection products (refer to Fig. 3).

The analysis of the results of the share of individual GHG emission sources in the total emission leads to an unequivocal conclusion that the distribution of CO_2_ eq emissions is differentiated for technological variants under study. When analyzing the results for 2019, it can be noticed that the greatest differences in the distribution occur in the “fertilizer use” group. The percentage share of this factor ranged from 57.3% for object B to 75.8% for object A (Figs. 1, 2, and 3). There are also noticeable differences in the share of GHG emissions related to the mineralization of crop residues. The highest share of this factor, amounting to 30.1%, was found in object B, where straw was left, and the lowest 8.1% in object A. In the case of emissions related to the production and use of biochar, its share in objects C–F ranged between 6.5 and 8.9%. The difference in the percentage share of the other two factors is not significant. Based on the independence test (χ^2^ = 441.94, *p* value = 0.000), it can be concluded that there is a significant variation in the distribution of the share of individual factors on CO_2_ eq emissions for the tested methods jointly. Additionally, based on the independence test, it was verified which distributions of the methods differ in pairs. As a result of this analysis, it was shown that both the distributions for method A and for method B differ from the distributions of all other methods. Moreover, the distribution in the C method significantly differs from the distributions obtained in the D and F methods. The results of the analysis carried out for the data from 2019 were repeated in the next 2 years, i.e., 2020 and 2021.

In the second step of the statistical analysis, to verify whether the average CO_2_ emissions for the production of 1 ton of corn differs significantly for individual methods, an analysis of variance was used for experiments with repeated measures. To verify whether it is possible to use the one-dimensional approach, the Mauchley sphericity test (1940) was performed. Its results are presented in Table 8.

**Table 8:** The results of Mauchly’s sphericity test

Result	W	Chi-sq	*df*	*p*
Year	0.872373	2.321146	2	0.313307

**Fig. 4:**
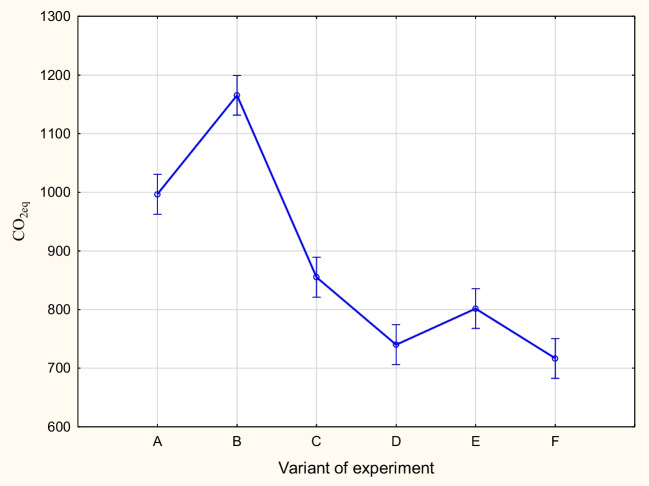
Graphical interpretation of the influence of the factor experiment variant on CO_2_ emission. Vertical bars represent the 95% confidence interval

**Fig. 5:**
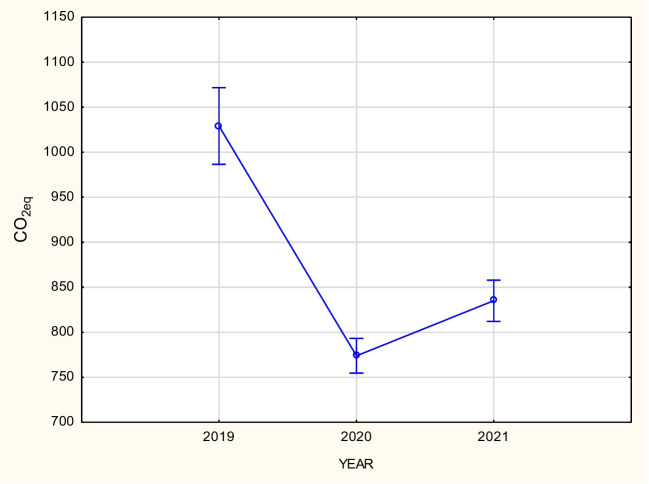
Graphical interpretation of the influence of the factor year of experiment on CO_2_ emission. Vertical bars represent the 95% confidence interval

As the test value is high and close to 1 (*W* = 0.872373), it entails the test probability level *p* = 0.0313307, which means that there are no grounds to reject the spherical hypothesis. Therefore, it is possible to use the one-dimensional approach. Due to the fact that there are two factors in the analysis, i.e., time and method, the mean boundary effects are presented in Figs. 4 and 5.

The results in the diagram related to the cultivation method (left) suggest that object A is the most unfavorable in terms of CO_2_ eq emissions per ton of corn produced. The average emission value for this method, for four plots over 3 years, is approximately 1180 kg CO_2_ eq/t of grains of corn. The lowest average CO_2_ eq emissions were obtained for methods F and D, and it was approximately 720–730 kg CO_2_ eq/t of corn grain (Figs. 4 and 5). The highest CO_2_ emission was observed in the first year of cultivation, i.e., 2019, and was 1030 kg of CO_2_ eq/t of grains of corn on average (for all objects). It was the lowest in the second year of cultivation, i.e., in 2020, at 775 kg of CO_2_ eq/t grain of corn.

**Fig. 6:**
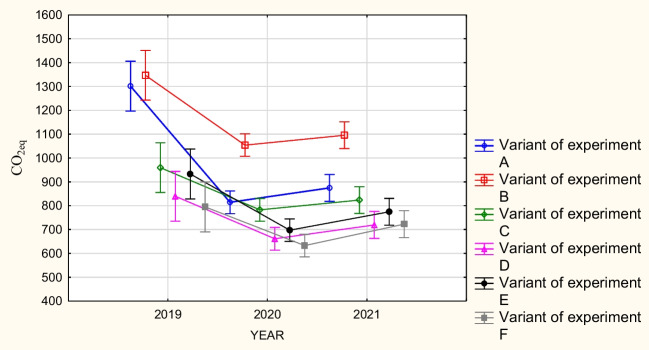
Graphical interpretation of the influence of the factors, experiment variant, and year of experiment/CO_2_ emission

Figure 6 shows the combined effect of method and time on average CO_2_ eq emissions. Average CO_2_ eq emission when using method B is the highest in each year compared to other methods. However, there is no method that is by far the best in terms of CO_2_ emissions. Namely, in 2019 and 2020, the lowest emission was obtained for method F and in the last year for method D. Method A gives a high emission result (similar to method B) in the first year, while in subsequent objects, the emission significantly decreases.

To verify the existing differences in the impact of factors, both individual and jointly, on the average CO_2_ eq emission, an analysis of variance with repeated measures was performed for the studied data. The main results of this analysis are presented in Tables 9, 10, and 11.

**Table 9:** Results of repeated ANOVA measures

Result	SS	*df*	MS	*F*	*p*
Free term	55665332	1	55665332	17780.47	0.000000
Method	1777428	5	355486	113.55	0.000000
Error	56353	18	3131		
Year	853525	2	426762	73.36	0.000000
Year * Method	214208	10	21421	3.68	0.001843
Error	209438	36	5818

**Table 10:** Scheffe’s test results for the “Variant of experiment” factor

Variant of experiment	A	B	C	D	E	F
A		0.000060	0.000510	0.000000	0.000009	0.000000
B	0.000060		0.000000	0.000000	0.000000	0.000000
C	0.000510	0.000000		0.004462	0.393675	0.000658
D	0.000000	0.000000	0.004462		0.255009	0.954633
E	0.000009	0.000000	0.393675	0.255009		0.051012
F	0.000000	0.000000	0.000658	0.954633	0.051012	

It turns out that both main effects, i.e., method and year, are statistically significant (*p* < 0.001), and their interaction effect is also significant (*p* < 0.01). Thus, the differences observed in the charts turned out to be statistically significant. This means that individual crop residue management strategies resulted in significant differences in CO_2_ eq emissions throughout the period considered. CO_2_ eq emissions differed significantly in individual years of cultivation (regardless of the method used). The type of method used differentiates the average emissions in each of the analyzed periods separately. The above results were obtained for the analysis of all methods and periods jointly. To verify which corn crop residue management strategies differ significantly from the others in terms of CO_2_ eq emissions, Scheffe’s post hoc test was carried out separately for the factors: “Variant of experiment” and “Year.”^1^

Table 10 gives grounds to conclude that both the average emissions for method A and for method B differ significantly from the average emission for other methods because all *p* values are lower than 0.05. The use of method C does not differentiate CO_2_ emissions only in relation to method E, while method D gives a result similar to E and F and significantly different from methods A, B, and C. Method E differs significantly only from A and B and method F additionally from C.

The Scheffe test results for the time factor presented in the table show that there are significant differences in the average amount of CO_2_ emissions in each of the analyzed periods (all probabilities are lower than 0.05, (Table 11). This means that in the second year of cultivation, the emission is significantly lower than in the first and third years, while in the third year, it increases significantly compared to the second year but is significantly lower than in the first year.

**Table 11:** Scheffe test results for the factor “year”

Year	2019	2020	2021
2019		0.000000	0.000000
2020	0.000000		0.030282
2021	0.000000	0.030282	

GHG emissions related to diesel combustion ranged from 107.3 to 149.4 kg of CO_2_ eq/t of produce. The share of this source of GHGs in individual research objects ranged between 9.2 and 17.2% (Figs. 1, 2, and 3). The emissions related to the use of mineral fertilizers had the highest share of total GHG emissions in all research objects. In each case, this value exceeded 50%. Regardless of the year of research, the highest share of this source was found in object A and the lowest in object B. When analyzing the absolute values, it was found that the highest value of emissions related to fertilizer use was found in objects A, B, and C and the lowest value in objects D, E, and F. The average value of GHG emissions related to the use of mineral fertilizers for all years was 719 CO_2_ eq/t in facility A, while in facility F, where the fertilizer produced on the basis of biochar and urea was applied, the value was 395.9 CO_2_ eq/t. In this study, CO_2_ eq emissions related to diesel combustion, the use of seeds and mineral fertilizers was between 2000 and 3000 kg/ha and was comparable to the data presented by Holka and Bieńkowski (2020). Qi et al. (2018) found that GHG emissions from corn cultivation range between 400 and almost 800 kg of CO_2_ eq/t depending on the level of intensification of production. However, these authors excluded the emissions related to the mineralization of crop residues and soil organic matter from the system boundary. Liu et al. (2021) report the values of the carbon footprint for corn cultivation in the Chinese province of Hebei at a higher level than that obtained in this research, i.e., approximately 2000 CO_2_ eq/t. These authors emphasize that in assessing the carbon footprint of agricultural systems, soil-bound carbon should be taken into account for a clearer approach to calculating the environmental impact of agriculture. N_2_O emissions from nitrogen unused by plants were a significant source of GHG emissions. Carbon dioxide emissions from soil are an important component of the total impact of plant production on the intensity of climate warming. The results of the own research show that the level of GHG emissions from soil ranged from about 1000 CO_2_ eq/ha to almost 4000 CO_2_ eq/ha. The highest values of this parameter were found in objects where straw was left in the field and the distribution of crop residues was the most important parameter influencing the GHG emission from this source. Chi et al. (2020) found CO_2_ emissions from the soil during the growing season of corn and wheat at 530 to over 600 kg when straw was left in the field. Assuming that the decomposition of organic matter also takes place in addition to the growth of plants, the total level of GHG emissions could be higher than estimated in this research. Pareja-Sánchez et al. (2019) found the annual amount of CO_2_ emissions from soil, in which corn was grown at approximately 1800–4500 CO_2_/ha. Large differences in carbon dioxide emissions resulted from the variable level of nitrogen fertilization. These authors emphasize the role of nitrogen as a factor that models carbon transformation in soil. Kumar et al. (2021) calculated the level of CO_2_ emissions from the soil in corn cultivation at approximately 1000–3000 kg of CO_2_/ha. These authors found a significant positive relationship between the level of nitrogen fertilization and the emission of carbon dioxide from the soil surface. In turn, Álvaro-Fuentes et al. (2016) found no unequivocal trend in CO_2_ emissions from the soil in the cultivation of unfertilized corn and fertilized with nitrogen at the dose of 300 kg N/ha. The values of emissions from the soil surface reported by these authors ranged from 4300 to 5600. Mdhluli and Harding (2021) prove that harvesting residual crop of corn and converting it for energy purposes could ensure better environmental effects than leaving it at the production site. These authors emphasize that residual crop of corn is poor in nitrogen and other macronutrients, and transforming it into highly efficient energy sources is the right direction of residual crop management. The use of residual crop after the torrefaction of organic matter could be more effective than introducing raw organic matter into the soil. However, many studies indicate that in the long term, such an approach will lead to a deterioration of soil fertility. The production potential of the habitat has the greatest impact on the level of GHG emissions from corn cultivation, which is related to the fertility of the soil and the production potential of the cultivated varieties. These parameters are not directly related to production technology, but in many cases play the greatest role in modeling the value of carbon footprint (Boone et al. 2016). Mineral fertilizers accounted for the largest share of GHG emissions in the presented research. For all facilities, except for the use of biochar fertilizers, the emission level from this source exceeded 3300 CO_2_ eq/ha. These values are comparable to those presented by Qi et al. (2018). The use of slow-release fertilizers in its own research resulted in a significant reduction in the share of this source in the total GHG emissions from corn production. When applied properly, slow-release fertilizers can significantly increase the efficiency of nitrogen fertilization, which reduces GHG emissions per plant yield (Sikora et al. 2020a, b). Leaving a residual crop at the plant production site is not the optimal way to manage carbon resources (Wang et al. 2021). Organic matter left in the form of mulch or mixed with soil undergoes intensive mineralization, which leads to the emission of significant amounts of carbon dioxide. In the authors’ own research, the mineralization of straw in the soil accounted for over 30% of the total GHG emissions related to corn production. In the remaining objects, this value was approximately 10% and was related to the mineralization of the roots and the uncollected part of the post-harvest residues. Cereal straw is not rich in nitrogen compounds; therefore, its decomposing biomass is not a significant source of this element. In intensive cultivation and high nitrogen fertilization, the humification processes are very limited, which means that the remaining straw is not a source of humus (Bei et al. 2022). Huang et al. (2021a) found that after 8 years of rice cultivation, the content of total organic carbon in the soil increased when straw was left in the field, compared to the objects with the straw removed. However, in the same studies, no statistically significant difference was observed in the accumulation of carbon in the soil between the objects where straw was left, and where straw was torrefacted and ash was left. The results presented by these authors prove the questionable value of straw as a permanent source of carbon bound carbon in the soil. The direction and intensity of organic compound transformations in soil are determined by many factors and is related to the complex ecological relations within the soil microbiome. Yuan et al. (2021) found an increase in the content of labile carbon fractions after 6 years of leaving wheat and rice straw in the field. However, the authors found that the permanent binding of organic carbon increased significantly when potassium was applied directly on the straw. This was accompanied by an increase in plant yield by approximately 20% compared to objects where straw was collected. Xie et al. (2021) emphasize that a critical element influencing the sequestration of carbon in straw left in the field is the right amount of available nitrogen and phosphorus. Too much nitrogen increases the level of intensification of organic matter mineralization, which is not beneficial from the point of view of carbon sequestration.

The available research results indicate that the environmental effectiveness of leaving straw at the cultivation site is determined by many factors related to production technology, soil properties, and climatic conditions. Therefore, it is not possible to provide unified recommendations on the management of harvest residues in light of specific environmental effects. Only the development of a technology to transform biomass into a more durable form and introduce it into the soil could provide the input data for the development of a methodology for the permanent sequestration of organic carbon in the soil. On the one hand, this would reduce the value of the carbon footprint of cultivated plants and, on the other hand, increase the level of carbon sequestration in the soil, improving its fertility.

The research results indicating the high environmental potential of the technology for converting organic waste into biochar could have a significant impact on the development of agriculture and environmental protection. Biochar, produced through the process of pyrolysis, which is the thermal decomposition of organic substances in the absence of oxygen, can be used as a soil additive that improves its structure, increases the content of organic carbon, and helps in carbon sequestration. The implementation of biochar in agriculture can bring the following benefits:

1. Increasing the content of organic carbon in the soil: Biochar is a stable source of organic carbon that can remain in the soil for decades, or even centuries, contributing to an increase in humus levels and improving soil quality.
2. Reducing greenhouse gas emissions: The use of biochar can help limit greenhouse gas emissions from agriculture through carbon sequestration in the soil and by replacing mineral fertilizers, which generate greenhouse gas emissions during their production and use.
3. Managing by-products: Using organic waste, such as straw or manure, for biochar production allows for more efficient management of by-products, reducing the need for burning or storage, which often leads to humus losses and greenhouse gas emissions.
4. Improving soil and crop quality: Biochar can improve the physical, chemical, and biological properties of the soil, which can lead to increased yields and improved quality of the crops grown.

However, the introduction of biochar into agricultural practice requires further research and testing to optimize the production and application processes under various soil and climatic conditions. Additionally, it is necessary to develop the infrastructure and technology needed for industrial-scale biochar production and to create methodologies and guidelines for farmers that will help them effectively use biochar in carbon farming. In the context of global efforts to mitigate climate change, biochar technology could become an important tool in actions for sustainable development and environmental protection.

The obtained research results constitute a very important element in enriching the knowledge regarding the specifics of carbon compound transformations in the soil, both in the context of carbon sequestration in agricultural soils and in the context of shaping soil fertility in the long-term perspective. Understanding these issues is crucial for creating principles of good agricultural practices under specific soil, climatic, and infrastructure conditions.

### Carbon sequestration

By understanding how carbon compounds are transformed and stored in the soil, researchers and practitioners can develop strategies to enhance carbon sequestration, which is vital for mitigating climate change. Knowledge about the long-term effects of different agricultural practices on soil fertility is essential for sustainable farming. With a better understanding of soil processes, farmers can be guided to adopt practices that are not only productive but also environmentally sustainable. As climate conditions change, so too will the requirements for maintaining soil health and fertility. Research on soil carbon dynamics can help in developing adaptive strategies that ensure food security and environmental resilience under changing climatic conditions. The research results on carbon transformations in the soil are a critical component for advancing our understanding of soil processes and for developing sustainable agricultural practices that can contribute to both food production and environmental stewardship.

The scientific literature emphasizes the potential of biochar in sequestering carbon in soil through the use of pyrolyzed organic matter, forming persistent organic compounds. To comprehensively evaluate the environmental impact of biochar, specific studies on its permanent carbon sequestration potential using distinct raw materials are crucial. Additionally, for enhanced organic carbon sequestration and reduced greenhouse gas emissions, exploring the incorporation of mineral additives to optimize biochar-based products is essential.

## Conclusion

The applied modifications of the corn cultivation technology in terms of the crop residue management strategy had a significant impact on the yield of plants and the amount of GHG emissions. The most unfavorable variant turned out to be leaving corn straw in the field in its raw form. The conversion of corn straw to biochar and its introduction into the soil reduced GHG emissions from corn cultivation per functional unit, despite the energy expenditure related to straw transport and biochar production. This was due to the reduction of carbon dioxide emissions associated with the mineralization of organic matter in the soil and an increase in the yield of plants in objects where biochar was used. In a 3-year time perspective, a beneficial effect of biochar addition on the size of the commercial yield of plants was observed. Removal of crop residue from the field had a positive effect on the level of GHG emissions related to the cultivation in the short term; however, a negative impact on the crop yield was observed already in the third year. Crop residue torrefaction and reintroduction into the soil positively impacted the efficiency of nitrogen fertilization. In the variants where biochar and a reduced level of nitrogen fertilization were used, no reduction in yields was observed. The biochar produced from corn straw turned out to be a valuable substrate for the production of organic and mineral fertilizers with the properties similar to slow-release fertilizers.

## Data Availability

Data and materials are not available.

## Footnotes

1 Due to the large size of the results table and difficult interpretation, the results of Scheffe’s test were omitted for both factors jointly.
